# Antitumor Immunity: Role of NK Cells and Extracellular Vesicles in Cancer Immunotherapy

**DOI:** 10.3390/cimb46010011

**Published:** 2023-12-25

**Authors:** Angelina E. Prokopeva, Charles C. Emene, Marina O. Gomzikova

**Affiliations:** 1 Laboratory of Molecular Immunology, Institute of Fundamental Medicine and Biology, Kazan Federal University, Kazan 420111, Russia; prokopeva_ae@mail.ru; 2 Laboratory of Intercellular Communication, Kazan Federal University, Kazan 420111, Russia; emene.charles@gmail.com

**Keywords:** extracellular vesicles, immune cells, natural killer cells, tumor cells, immune response, immunotherapy, cellular immunotherapy, CAR-NK cells, recognition, destruction

## Abstract

The immune system plays a crucial role in recognizing and eliminating altered tumor cells. However, tumors develop mechanisms to evade the body’s natural immune defenses. Therefore, methods for specifically recognizing/targeting tumor cells, for instance, through the activation, directed polarization, and training of immune cells, have been developed based on the body’s immune cells. This strategy has been termed cellular immunotherapy. One promising strategy for treating tumor diseases is NK cell-based immunotherapy. NK cells have the ability to recognize and destroy transformed cells without prior activation as well as tumor cells with reduced MHC-I expression. A novel approach in immunotherapy is the use of extracellular vesicles (EVs) derived from NK cells. The main advantages of NK cell-derived EVs are their small size and better tissue penetration into a tumor. The aim of this review is to systematically present existing information on the mechanisms of antitumor immunity and the role of NK cells and extracellular vesicles in cancer immunotherapy. Clinical and preclinical studies utilizing NK cells and extracellular vesicles for anticancer therapy currently underway will provide valuable insights for researchers in the field of cancer.

## 1. Introduction

Current WHO figures show that cancer is one of the leading causes of death worldwide, and the frequency of new cancer cases detected increases each year [1]. When tumor cells appear, the immune system protects the human body by eliminating these cells. Recognition of tumor cells occurs via a series of biochemical markers that differentiate them from normal cells in the body. Nevertheless, some tumors develop mechanisms that allow them to evade immune surveillance. The process of interaction between the immune system and the tumor is described by the hypothesis of immune editing and consists of three phases:

1. Elimination phase: Cells that have accumulated mutations are detected and eliminated by the immune system. This is possible because cells begin to express stress ligands and tumor-associated antigens. This results in the activation of antitumor immunity [2].
2. Equilibrium phase: Adaptive immune resistance is established in this phase [3]. This is characterized by immune selection by T-cells of tumor cell clones lacking expression of rejection antigens. Tumor cells that have not been recognized and eliminated persist in the body without progressing [3,4]. Mechanisms facilitating immune evasion and cell survival involve epigenetic suppression of highly immunogenic tumor-associated antigens [3,5].
3. Evasion Phase: Cancer cells can grow and metastasize while avoiding detection by the immune system due to the genetic and epigenetic changes that accumulate during the equilibrium phase. These cancer cells suppress the immune response by expressing various molecules, such as immune checkpoint ligands, which in turn reduce the likelihood of being recognized by immune cells [6,7].

Traditional methods for treating malignant tumors, such as chemotherapy and radiation therapy, come with significant side effects and can lead to the development of resistance, diminishing their effectiveness. Consequently, immunotherapy has emerged as a promising approach to treatment. This approach is based on the body’s natural defense mechanisms against transformed cells. Therefore, it has minimal side effects. There are several approaches to immunotherapy presently practiced, with cellular immunotherapy being a notable one. This approach involves the adoptive transfer of activated/modified cells. In this review, our focus is on the challenge of developing new methods for anticancer immunotherapy based on immune cells. Understanding the mechanisms behind the antitumor response of immune cells and the role of NK cells and extracellular vesicles will enable the development of novel strategies for cancer immunotherapy.

## 2. Antitumor Immune Response Mechanism

The human immune system plays a crucial role in protecting the body by detecting, recognizing, and eliminating foreign agents [8]. It is known that the immune response is classified into innate and adaptive (acquired) immunity. Innate immunity is nonspecific and provides a rapid primary response after antigen penetration. Cells of the innate immune system include dendritic cells, macrophages/monocytes, mast cells, granulocytes, and natural killer (NK) cells. Conversely, the adaptive immune response is specific. Cells of the adaptive immune system encompass T-lymphocytes and B-lymphocytes. As a result of the adaptive immune response, immune memory is formed, which is necessary for a rapid and specific immune response upon reencountering a previously eliminated antigen. The combined action of innate and adaptive immune responses safeguards the body against foreign pathogens and damaged and malignantly transformed cells [9].

**Figure 1:**
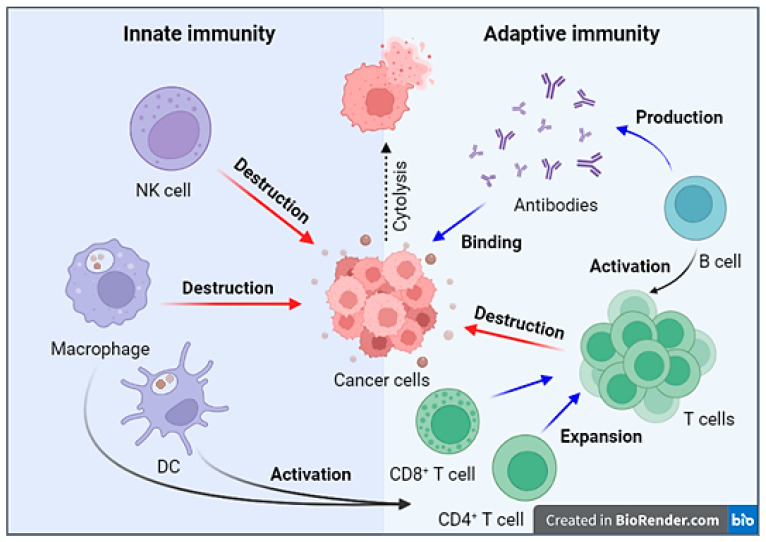
The interaction of immune system components in response to tumor cells. When tumor cells are detected, NK cells initiate the destruction of tumor cells through direct interaction. Macrophages perform phagocytosis of tumor cells and, along with dendritic cells (DC), serve as antigen-presenting cells (APCs) presenting tumor antigens as part of the MHC complex on their membrane, thereby activating T-cells. Cytotoxic T-cells proceed to eliminate tumor cells. B-cells also function as APCs and have the capacity to activate T-cells. B-cells secrete antibodies, which mediate ADCC and ADCP. Created with BioRender.com (accessed on 2 October 2023).

On detection of a transformed cell, a series of immune processes occur, which can be briefly described in the following sequence. In the initial stage, the first line of defense is activated, which involves cells of the innate immune system. NK cells interact directly with tumor cells, recognizing ligands on their surface and exhibiting cytotoxic activity [8]. Monocytes, under the influence of cytokines, differentiate into macrophages, which phagocytize tumor cells [7]. The activity of innate immune cells, accompanied by the destruction of tumor cells and the capture of tumor antigens by antigen-presenting cells (APCs), initiates the adaptive immune response [10]. The role of APCs is carried out by dendritic cells and macrophages/monocytes, which recognize and engulf tumor antigens and subsequently present processed antigen fragments bound to the major histocompatibility complex (MHC) on their surfaces [10]. APCs then migrate to secondary lymphoid organs, where the recognition of the antigen–MHC complex on the surface of APCs leads to the activation of naive T-lymphocytes [11]. Cytotoxic CD8+ T-lymphocytes recognize antigen fragments bound to MHC-I, leading to the activation and expansion of cytotoxic T-lymphocytes that destroy tumor cells [10]. Meanwhile, CD4+ helper T-cells recognize the antigen–MHC-II complex [12]. CD4+ helper T-cells contribute to maintaining effector functions and the differentiation of CD8+ T-lymphocytes through cytokine production [13]. B-lymphocytes also function as APCs and facilitate the expansion of both CD4+ and CD8+ T-lymphocytes by presenting internalized antigen fragments as part of the MHC complex. This starts with B-lymphocytes presenting antigen fragments bound to MHC-II to CD4+ T-lymphocytes. This also serves as co-stimulatory signals for B-cell activation enabling the activated B-lymphocytes to acquire an enhanced antigen-presenting capacity [14,15]. Upon stimulation by tumor antigens, B-lymphocytes differentiate into plasma cells that secrete antibodies capable of recognizing tumor antigens on the surface of tumor cells and binding to them [16]. Antibodies are recognized by NK cells and macrophages due to the presence of Fc receptors (FcRs) on their surfaces. This recognition triggers mechanisms of antibody-dependent cellular cytotoxicity (ADCC) by NK cells and antibody-dependent cellular phagocytosis (ADCP) by macrophages [16]. Following ADCP of target cells, antigen fragments are presented as part of the MHC complex on the surface of macrophages, leading to the activation of T-lymphocytes [17] (Figure 1).

Cells of the immune system recognize tumor-specific antigens (TSAs) and tumor-associated antigens (TAAs) expressed by tumor cells. Tumor-specific antigens are found exclusively in tumor cells, while tumor-associated antigens are expressed in healthy cells but have an elevated level of expression in tumor cells [18]. Cells of the immune system recognize and eliminate altered cells; however, over time (in the evasion phase), the tumor develops mechanisms that enable it to evade the immune system. One of the mechanisms of tumor evasion involves inducing immune checkpoint ligands on the surface of tumor cells. These ligands hinder the activation of immune cells and their antitumor activity [19]. Furthermore, mutations in tumor cells can lead to the loss of MHC-I expression, reducing immune recognition and promoting the tumor’s evasion of immune surveillance [20,21]. Additionally, immune cells less effectively recognize tumor-associated antigens (TAAs) on tumor cells due to the development of self-tolerance [21]. A new therapeutic strategy aimed at overcoming the evasion of tumor cells from the immune system is immunotherapy. It encompasses the following approaches: oncolytic viral therapy, cytokine therapy, the use of anticancer vaccines, adoptive cell transfer (cellular immunotherapy), and the utilization of immune checkpoint inhibitors [12]. In this review, we have placed particular emphasis on cellular immunotherapy, which has emerged as a promising direction in cancer therapy. Cellular immunotherapy, or adoptive cell transfer (ACT), involves the infusion of immune effector cells that have been preactivated and/or modified and expanded ex vivo [12,22].

## 3. NK Cells: Mechanism of Action

The inherent ability of NK cells to recognize and eliminate transformed cells without prior activation makes them attractive tools in cancer therapy. NK cells are a subset of innate lymphoid cells within the immune system, and they possess cytotoxic activity against infected and transformed cells. In addition to their cytotoxic activity, NK cells secrete cytokines such as IFNγ, TNF, IL13, CCL3, CCL4, and CCL5 [23]. When combined with the cytotoxic activity of NK cells, cytokine secretion enhances the antitumor immune response [10].

NK cells are found in the blood, lymph nodes, lungs, liver, kidneys, thymus, and uterus [24]. Markers for detecting NK cells include the expression of neural cell adhesion molecule (NCAM) (CD56) and low-affinity immunoglobulin gamma Fc region receptor IIIA (FCγRIIIA) (CD16). Since CD56 and CD16 are found on some populations of T-lymphocytes, an additional marker, TCR/CD3, which is a marker for T-cells and is not present on the surface of NK cells, is included to specifically detect NK cells [23].

NK cells make up approximately 10% of peripheral blood mononuclear cells [25]. The majority of peripheral blood NK cells are characterized by low expression of CD56 and high expression of CD16 and exhibit more pronounced cytotoxic activity compared to NK cells with high CD56 and low CD16 expression. The latter group represents only a small population of peripheral blood NK cells, but these cells are strong cytokine producers [26].

### 3.1. The Recognition Mechanism

NK cells express both activating and inhibitory surface receptors. Some of the most significant activating receptors include natural cytotoxicity receptors (NCRs), killer cell immunoglobulin-like receptors (KIRs) such as KIR-2DS and KIR-3DS, and lectin-like receptors NKG2, specifically NKG2D and NKG2C/CD94. Inhibitory receptors include NKG2A/CD94, KIR-2DL, and KIR-3DL. Inhibitory receptors play a crucial role in ensuring tolerance toward healthy self-cells. Conversely, activating receptors can recognize ligands associated with tumor activity present on the surface of cells [23,27].

When activating receptors interact with tumor ligands, they bind to adapter proteins containing immunoreceptor tyrosine-based activation motifs (ITAMs). These adapter proteins primarily include DAP12, FcRγ, DAP10, and CD3ζ. Subsequently, ITAM adapters undergo phosphorylation by SFK kinases, initiating a cascade of reactions, leading to the induction of the PAK1-MEK-Erk signaling pathway and the MAPK pathway. Additionally, DAP10 can bind to p85 PI3K and the Grb2-Vav-1-SOS1 complex, activating Akt/PKB. Ultimately, this results in the activation of NK cell cytotoxicity.

Inhibitory receptors have intracellular domains containing immunoreceptor tyrosine-based inhibition motifs (ITIMs). Upon receptor recognition of ligands, ITIM sequences undergo phosphorylation by SFK kinases, initiating a cascade of dephosphorylation reactions involving phosphatases, primarily SHP1 and SHP2. Inhibitory receptors induce the dephosphorylation of Vav1, LAT, and phospholipase C 1/2 (PLCγ1/2). This leads to the suppression of NK cell activation signals. Additionally, a mechanism inhibiting NK cell function has been identified, based on the phosphorylation of Crk upon binding to the Abl tyrosine kinase, which is crucial for separating Crk from the Cb1-Crk-C3G complex, a part of the activating signaling pathway [27].

The regulation of NK cell activity relies on the net sum of signals from activating and inhibitory receptors that interact with their respective ligands on target cells. If the sum of signals from activating receptors exceeds the sum of signals from inhibitory receptors, NK cells become activated, leading to the destruction of the target cell [27,28].

However, CD16 is capable of activating NK cells without the need for additional activation from other receptors [28]. This occurs through the expression of the CD16A molecule, which recognizes the Fc portion of immunoglobulin bound to antigens on the surface of target tumor cells, initiating the ADCC mechanism [29]. During this process, intracellular ITAM within the CD16A receptor in NK cells becomes phosphorylated, leading to NK cell activation [30].

Organism cells express peptides of their own proteins as part of the MHC complex, which is necessary for the identification of altered cells by immune cells. If a cell contains foreign peptides, it is eliminated [2]. Many cancer cells tend to reduce the expression of MHC-I molecules, allowing them to evade the host’s immune system. At the same time, MHC-I molecules act as ligands for the inhibitory receptors of NK cells. The decreased expression of MHC-I in tumor cells leads to a reduction in the signal from inhibitory receptors and, ultimately, an excess of signals from activating receptors on NK cells. As a result, one distinctive feature of NK cells is their ability to recognize and destroy tumor cells with reduced MHC-I expression [27,28]. Since NK cells do not respond to foreign MHC molecules, this opens up new possibilities for the use of allogeneic NK cell adoptive therapy as an alternative to T-cells, minimizing the risk of graft-versus-host reactions [25].

### 3.2. The Destruction Mechanism

**Figure 2:**
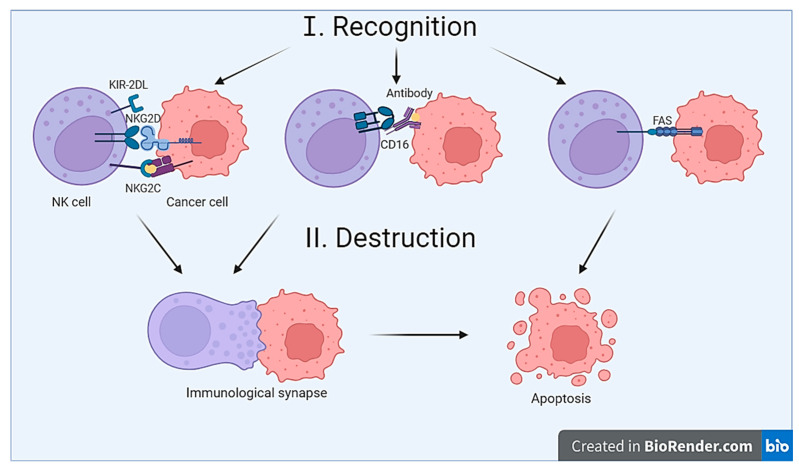
Mechanisms of recognition and destruction of tumor cells by NK cells. Tumor cell recognition occurs through three distinct mechanisms: (1) summation of signals from activating and inhibitory receptors, (2) interaction of CD16 on the NK cell with immunoglobulin on the surface of the tumor cell, and (3) interaction of the ligand (FasL) on the NK cell with the death receptor on the surface of the tumor cell. Tumor cell destruction occurs through two pathways: (1) via the formation of an immunological synapse and (2) through initiation of death receptor-mediated apoptosis. Created with BioRender.com (accessed on 2 October 2023).

There are several mechanisms through which NK cells can destroy target cells (Figure 2):

1. The mechanism of target cell destruction is possible due to the presence of cytolytic granules in NK cells [29]. After recognition and contact with the target cell, NK cell activation occurs, leading to the formation of the so-called immunological synapse. The immunological synapse represents the contact (or gap) between the NK cell and the target cell [31]. Inside the NK cell, cytolytic granules are transported toward the synapse. Subsequently, cytolytic granules merge with the cell membrane, resulting in the release of their contents into the synaptic cleft [32]. Cytolytic granules contain perforin, granzymes, granulysin, FasL (CD178), and TRAIL (CD253) [8]. After being released from cytolytic granules, perforin penetrates the target cell’s membrane, oligomerizes, and forms pores in the membrane [33]. The pore-forming activity of perforin depends on its polymerization, pH, and the presence of Ca^2+^.
  Granzymes, which are serine proteases, enter through the pores formed by perforin, inducing target cell apoptosis through both caspase-dependent and caspase-independent mechanisms. Granzyme A induces caspase-independent apoptosis by cleaving histones and affecting the SET complex, making DNA accessible to cellular nucleases. Additionally, by affecting mitochondria, granzyme A leads to the accumulation of reactive oxygen species, which also damages DNA. Granzyme B activates initiator caspases, facilitates the release of cytochrome C from mitochondria, and can directly affect effector caspases 3 and 7, ultimately leading to cell apoptosis [8].
  Granulysin belongs to the saposin-like protein family and possesses pore-forming activity. Granulysin induces ion fluxes—intracellular calcium levels increase, while potassium levels decrease. This contributes to mitochondrial damage, the release of cytochrome C, and the activation of effector caspases, ultimately leading to target cell apoptosis. Additionally, granulysin is capable of damaging the ER and activating caspase 7, also leading to apoptosis induction [8,32]. Thus, the activation of NK cell cytotoxicity involves the recognition and contact with the target cell, the formation of the immunological synapse, and the release of cytolytic granules necessary for target cell lysis.
2. NK cells are capable of inducing receptor-mediated apoptosis of target cells [29]. Ligands for death receptors include TNF, FasL, and TRAIL. For example, FasL expressed by NK cells can bind to the Fas receptor (CD95) on the target cell’s membrane. As a result, the assembly of the death-inducing signaling complex (DISC) occurs within the target cell, leading to caspase activation and, ultimately, target cell apoptosis [32].

## 4. The Application of NK Cells in Antitumor Therapy

The promising potential of using NK cells as a therapy for treating oncological diseases has been demonstrated in several preclinical and clinical studies [34,35,36,37,38]. Current clinical trials involving NK cells are in Phase I/II of clinical trials (Table S1, Supplementary Materials).

In one study, the clinical efficacy and safety of percutaneous cryoablation in combination with allogeneic NK cell immunotherapy were evaluated in patients with advanced non-small cell lung cancer (NSCLC). The therapy demonstrated a synergistic effect, improving the antitumor outcome compared to cryoablation alone and showing favorable outcomes for patients. The study confirmed the safety and effectiveness of this combined therapy [39].

In another study, the safety of adoptive immunotherapy using activated NK cells was assessed in patients with refractory/relapsed acute myeloid leukemia (AML). None of the patients (7 individuals) experienced dose-limiting toxicity, indicating the safety of this approach [40].

In a study by Li et al., it was demonstrated that adoptive NK cell therapy in combination with chemotherapy prevented recurrence and prolonged the survival of patients with locally advanced colorectal cancer, with minimal side effects (bone marrow suppression, nausea, vomiting, and fever). The 5-year progression-free survival and overall survival were higher in the NK cell therapy combined with chemotherapy group compared to the control group (treatment with chemotherapy alone) [35].

In many studies, the NK-92 cell line has been used as a source of NK cells; this cell line represents a population of cells derived from peripheral blood mononuclear cells from a 50-year-old, White male with rapidly progressive non-Hodgkin’s lymphoma. Using NK-92 cells allows for a potentially less costly production of NK cell-based preparations; however, there are limitations associated with the fact that NK-92 cells have unlimited division capacity. Prior to administration to the patient, lethal irradiation of the cells is necessary, which reduces their further survival in the patient’s blood [41]. Additionally, NK-92 cells lack expression of the CD16 molecule, making them incapable of antibody-dependent cell-mediated cytotoxicity, thereby reducing their cytotoxic activity against tumor cells [26].

However, despite all the advantages and effectiveness of using NK cells for cancer immunotherapy, there are some limitations. The functional activity of NK cells can be suppressed by immunosuppressive molecules produced by immune cells in the tumor microenvironment and tumor cells, such as IL-10, TGFβ, and prostaglandin E2. Conditions of hypoxia, which are characteristic of solid tumors, also reduce the cytotoxic activity of NK cells [28]. Additionally, tumor cells can downregulate the expression of ligand molecules that interact with activating receptors on NK cells. All of these factors drive the search for new approaches and methods for NK cell-based cancer immunotherapy.

### Genetic Modification of NK Cells

One way to enhance the natural cytotoxicity of NK cells is through their genetic modification with a chimeric antigen receptor (CAR) capable of directing NK cells towards tumor cells [29]. A CAR is an engineered receptor protein consisting of an extracellular domain, a transmembrane domain, and an intracellular domain [42]. The extracellular domain of CAR binds to the target antigen located on the surface of tumor cells. The transmembrane domain anchors CAR to the cell membrane and connects it to the intracellular domain, which is responsible for activating the CAR-NK cell by providing an activation signal [29]. Often, a single-chain variable fragment (scFv) of an antibody specific to the antigen is used as the antigen-binding extracellular domain [43]. The target antigens can include molecules expressed on the surface of tumor cells, such as CD7, CD19, and CD33 [44]. Therefore, CAR-NK cells recognize the target antigen and eliminate antigen-expressing tumor cells [45].

Initially, constructions designed for CAR-T cells were used to modify NK cells. However, since NK cells have their own activating and inhibitory receptors, it is possible to incorporate into the CAR design intracellular domains specific to NK cells, such as 2B4, DAP10, and DAP12, with the aim of enhancing the cytotoxicity of NK cells [26].

CAR-NK cells have several advantages compared to CAR-T cells. Firstly, they are safer because they are less toxic to normal tissues and cause fewer events related to cytokine release syndrome and neurotoxicity. Secondly, given the wide range of receptors on the surface of NK cells, CAR-NK cells can exhibit cytotoxicity against a heterogeneous tumor wherein not all cells express the CAR antigen. Thirdly, due to the reduced risk of graft-versus-host reaction, there is the possibility of allogenic CAR-NK cell administration, allowing for their production on a large scale using various sources of NK cells such as peripheral blood mononuclear cells, NK-92 cells, induced pluripotent stem cells, and umbilical cord blood cells [26]. In the study by Liu et al., it was shown that the use of genetically modified CAR-NK cells was not associated with the development of cytokine release syndrome, graft-versus-host reaction, or an increase in inflammatory cytokine levels [46]. The reason for this is that NK cells, unlike T cells, do not react to foreign MHC, thus avoiding an immune response during transplantation, which is an advantage.

Currently, research is underway on CAR-NK cell therapy for hematological malignancies and solid tumors, with studies in the early phases of clinical trials (Phase I/II). In one of the studies, NK cells were transduced with a retroviral vector expressing genes encoding CAR-CD19, interleukin-15, and an inducible caspase-9-based suicide gene (iC9). In preclinical studies, the authors demonstrated that NK cells efficiently killed CD19-expressing cells and primary leukemia cells in vitro, leading to a marked prolongation of survival in a xenograft Raji lymphoma murine model. Additionally, the production of interleukin-15 (IL-15) by the transduced NK cells significantly improved their function [47]. Results from the clinical trial demonstrated that the use of iC9/CAR.19/IL-15 NK cells targeting CD19-positive lymphoid malignancies is safe and effective in patients with CD19+ CLL and B-cell lymphoma [46].

However, despite the achievements made, further research on CAR-NK cells is needed to overcome challenges such as tumor cell heterogeneity, the immunosuppressive tumor microenvironment, which can negatively affect CAR-NK cells, and the loss of the target antigen [26]. Additionally, NK cells have limited ability to penetrate solid tumor tissues [29]. Therefore, new approaches and anti-tumor tools are being developed that will not be susceptible to the reprogramming influence of the tumor microenvironment and will have better penetration capabilities. One such tool could be extracellular vesicles (EVs) derived from NK cells [29].

## 5. NK Cell Vesicles for Cancer Therapy

EVs, or extracellular vesicles, are nano-sized extracellular vesicles enclosed by a lipid bilayer. They are naturally secreted by cells and serve as mediators of intercellular communication between donor and recipient cells.

EVs encompass exosomes, microvesicles, and apoptotic bodies. Exosomes typically have a diameter ranging from 40 to 100 nm. They are formed from early endosomes that give rise to multivesicular bodies. Upon fusion with the plasma membrane, exosomes are released [48]. Microvesicles, on the other hand, range in size from 50 to 1000 nm and are generated through budding from the cell membrane [49]. Apoptotic bodies, which measure between 50 and 5000 nm, are formed as a result of programmed cell death, known as apoptosis [29]. These vesicles play a critical role in facilitating various cellular processes and intercellular signaling.

EVs (extracellular vesicles) contain typical markers characteristic of NK cells, such as CD56, along with cytotoxic proteins like perforin, granzymes, and granulysin. As a result, they possess the capability to induce apoptosis in tumor cells, similar to their parent cells [8,29]. Furthermore, it has been demonstrated that extracellular vesicles (EVs) carry cytokines and microRNAs [50] and exhibit biological activity similar to that of parent cells [51]. In a study by Neviani et al., it was shown that exosomal miR-186 directly suppresses MYCN and AURKA in neuroblastoma cells, potentially exerting a negative impact on their survival. Transfection of NK cells with the tumor suppressor miR-186 led to the downregulation of TGFBR1 and TGFBR2, thereby preventing TGFβ1-dependent inhibition of NK cell cytotoxicity [52]. As demonstrated in the study by Federici et al., EVs contain a significant amount of various soluble factors such as IL-8, CD62L, IL-2, IL-6, and IFNγ, indicating that EVs can stimulate other immune cells to enhance the anti-tumor response [53]. Additionally, in the work by Dosil et al., it was shown that EVs from NK cells contain microRNAs that influence the function of T cells, promoting Th1-like responses marked by increased release of IFN-γ and IL-2. Moreover, NK-EVs influence monocytes, stimulating their activation and enhanced presentation, providing an additional anti-tumor effect [54].

EVs offer a means of circumventing the limitations associated with the use of NK cells. Due to their nanoscale size, they exhibit superior penetration into tumor tissue. Moreover, the acidic microenvironment within solid tumors facilitates better fusion between EVs and tumor cells. EVs can be employed in the treatment of central nervous system tumors, as they can traverse the blood-brain barrier. Additionally, EVs can be stored at −80 °C for up to 12 months [29]. These advantages position EVs as a promising avenue for cancer therapy, addressing some of the challenges associated with NK cell-based approaches.

The mechanism of cytotoxic action of EVs closely resembles the mechanisms described earlier for NK cells. Upon fusion of EVs with the membrane of the target cell, perforin, granzymes, and granulysin are activated, while death ligands may interact with death receptors on the target cell membrane, leading to apoptosis [8]. The content of cytotoxic proteins inside EVs depends on their cellular source. In a study conducted by Chun-Hua Wu et al., it was demonstrated that EVs derived from ex vivo expanded NK cells primarily have higher levels of cytotoxic proteins compared to EVs derived from NK-92 cells [55]. This variance in cytotoxic protein content could impact the efficacy of EVs in cancer therapy depending on their source.

To date, EVs have been successfully used in preclinical studies for the treatment of malignant neoplasms. Their cytotoxic effects have been demonstrated against malignant hematological cells, neuroblastoma cells, melanoma, breast carcinoma, colorectal cancer, liver cancer, gastric cancer, ovarian cancer, lung cancer, and cervical cancer [55,56,57,58,59,60,61,62]. The anti-tumor activity of EVs has been confirmed in in vivo studies using mouse models with xenotransplants of melanoma [59], hepatocellular carcinoma [62], and breast cancer [58].

In a study by Wu et al., it was demonstrated that NK-derived extracellular vesicles (NK-EVs) have high levels of perforin and granzyme A, moderate levels of granulysin and granzyme B, and a low amount of FasL. Thus, the combination of cytotoxic proteins contributes to the cytotoxicity of NK-EVs. Moreover, it was shown that NK-EVs can cleave SET and HMG2, promoting target cell death through a caspase-dependent apoptotic pathway and inducing the release of cytochrome C from mitochondria in target cells [55]. However, information on how NK-EVs interact with tumor cells is limited. Drawing on numerous studies of the interaction of vesicles from human cells with target cells [63], it is presumed that the interaction occurs through plasma membrane fusion [64], clathrin-mediated endocytosis, or receptor-mediated internalization [65].

In one study, EVs were obtained using NK-92 cells as the cell source, and their anti-tumor activity was tested in a mouse model with melanoma xenotransplants. A total of 12 mice were used in the study. Half of the mice received intratumoral EVs, while the other half received PBS (control group). The progression of tumor growth was monitored. As a result, it was shown that EVs inhibited tumor growth and reduced tumor volume compared to the control group [54]. In a murine model with orthotopic xenotransplants, NK-92 EVs suppressed tumor growth in a dose-dependent manner compared to the control [62].

Choi et al. investigated the anti-tumor activity of EVs obtained from human NK cell-enriched lymphocytes. The authors demonstrated in vivo, using a mouse model (8 mice) with breast cancer xenotransplants, that EVs suppressed tumor growth compared to the control group. The final tumor mass was significantly smaller in the EV group than in the control group [58].

To enhance vesicle production, NK cells are treated with cytokines. In the study by Dosil et al., it was found that culturing NK cells in the presence of IL-12 and IL-18, followed by the addition of IL-2, leads to an increased release of vesicles [54]. In the study by Zhu et al., it was shown that NK cells, pre-activated with IL-15, produced a greater quantity of EVs compared to non-activated IL-15 NK cells. Furthermore, they exhibited stronger cytotoxicity against tumor cells, including glioblastoma, thyroid cancer, and breast cancer. In addition, the authors demonstrated the effectiveness of using EVs obtained from activated IL-15 NK cells in a mouse model with glioblastoma xenotransplants. Importantly, no cytotoxicity of EVs against healthy cells was observed [66].

These results demonstrate that the use of EVs derived from NK cells offers new advantages compared to cell-based therapy and is a promising approach for the immunotherapy of malignant diseases. The next step towards licensing an anti-tumor product based on NK cell-derived EVs is the development of an industrial-scale production method that ensures the following parameters: integrity, homogeneous size, absence of aggregates, absence of xenogenic contaminants, low levels or absence of chemical contaminants, and cellular components (nuclear DNA, organelles, proteins) [67].

## 6. Conclusions

Cellular immunotherapy is an effective approach in cancer treatment, but it exhibits modest anti-tumor activity. Immune-related adverse events, reprogramming of immune cells in the tumor microenvironment, and limited tumor infiltration are limiting factors. Therefore, there is a need to develop new tools for anti-tumor therapy based on genetically modified immune cells and extracellular vesicles (EVs) derived from immune cells. NK cells are particularly well-suited for these purposes as they serve as the first line of defense in the body, have advantages such as not requiring prior activation, absence of immune-related adverse events, and the ability to recognize and destroy tumor cells with reduced MHC-I expression.

The use of NK cell-derived EVs allows for the preservation of all the advantages of the parental cells while overcoming their limitations, such as limited tumor infiltration. EVs are smaller in size compared to the parental cells and demonstrate good biodistribution in vivo. EVs derived from NK cells have already shown promise as a tool for treating cancer in preclinical trials both in vitro and in vivo using animal models. However, at the time of writing this article, no clinical trials using EVs derived from NK cells were found in the clinical trials database. This is likely due to the low yield of EVs and the labor-intensive procedure for their isolation. Therefore, the current focus of research is on developing methods for the large-scale production of extracellular vesicles.

## Data Availability

All data generated and analyzed during this study are included in this published article. The data that support the findings of this study are available from the corresponding author upon request.

## Supplementary Materials

The following supporting information can be downloaded at: https://www.mdpi.com/article/10.3390/cimb46010011/s1.
