# Supplementary material for: Antitumor Immunity: Role of NK Cells and Extracellular Vesicles in Cancer Immunotherapy
Source: Curr Issues Mol Biol. 2023 Dec 25;46(1):140–52. doi: 10.3390/cimb46010011 (PMC10814167; doi:10.3390/cimb46010011)
Supplement: Supplementary file 1 [file cimb-46-00011-s001.zip › cimb-2697048-supplementary.pdf]

Table S1. Current clinical trials involving NK cells are in Phase I/II of clinical trials.

Intervention/treatment	Cell Dosage/Conditions	Cell source	Estimated Enrollment	Disease	First Posted	Identifier	Phase	Organization, country
NK cells	Infusion of 1-2×10^9 NK cells every 14 days	autologous	120	Small Cell Lung Cancer	January 25, 2018	NCT03410368	II	The First Hospital of Jilin University, China
PD-L1 CAR NK cells combined with pembrolizumab and N-803	Cells (2x10^9) will be administered by IV infusion over approximately 30 minutes every week	allogeneic	55	Gastroesophageal Junction (GEJ) Cancer; Advanced HNSCC	April 19, 2021	NCT04847466	II	National Institutes of Health Clinical Center (CC) (National Cancer Institute (NCI)), United States

Infusion of itNK cells	Induced T-to-natural killer (ITNK) cells	autologous	30	Cancer with lack of MHC-I	March 20, 2019	NCT03882840	I/II	Second Affiliated Hospital of Guangzhou Medical University, China
Hsp70-peptide TKD/IL-2 activated NK cells	NK cells after radiochemothe rapy	autologous	90	NSCLC Stage IIIA/B	April 21, 2014	NCT02118415	II	Technical University of Munich, Germany
Irreversible electroporation + NK immunotherapy	–	–	60	Pancreatic Cancer	March 24, 2016	NCT02718859	I/II	Fuda Cancer Hospital, Guangzhou; China
BiCAR-NK cells (ROBO1 CAR-NK cells)	–	–	9	Relapsed and refractory pancreatic cancer of ROBO1 expression	May 8, 2019	NCT03941457	I/II	Asclepius Technology Company Group (Suzhou) Co., Ltd., China

Trastuzumab + NK cells	Patient will receive intravenous trastuzumab, subcutaneous IL-2, NK cell infusion, followed by subcutaneous IL-2 for an additional 5 doses three times a week	autologous	29	HER2-positive breast or gastric cancer	January 8, 2014	NCT02030561	I/II	National University Hospital, Singapore
High-activity natural killer	Each treatment: 8~10 billion cells in all, transfuion in 3 times, i.v.	-	20	Non-small Cell Lung Cancer Metastatic	January 2, 2017	NCT03007875	I/II	Fuda Cancer Hospital, Guangzhou, China
			20	Metastatic Colorectal Cancer		NCT03008499		
			20	Pancreatic Cancer		NCT03008304		
			20	Metastatic Nasopharyngeal Cancer		NCT03007836		
			20	Tongue Cancer		NCT03008330		

Decitabine combined with NK cell	Decitabine combined with NK cell infusion as post-remission therapy	–	60	Malignancy	December 3, 2021	NCT05143125	I/II	Shenzhen University General hospital, China
Combination of cryosurgery and NK immunotherapy	Each treatment: about 10 billion cells transfusion in 3 times, i.v.	–	20	Liver Tumor Evidence of Liver Transplantation	July 29, 2016	NCT02849015	I/II	Fuda Cancer Hospital, Guangzhou, China
			30	Secondary Malignant Neoplasm of Liver	July 26, 2016	NCT02843802		
			30	Metastatic Renal Cell Cancer		NCT02843607		
			60	Breast Cancer Recurrent		NCT02844335		
			30	Non-small Cell Lung Cancer Metastatic		NCT02843815		
Combination of cryosurgery and NK immunotherapy	Natural killer cell immunotherapy for more than 6 times	–	60	Metastatic Esophageal Cancer	July 26, 2016	NCT02843581	I/II	Fuda Cancer Hospital, Guangzhou, China

Activated and expanded natural killer cells (NKAEs)	–	haploidentical donor	13	Relapsed/Refractory Paediatric Acute Leukaemia	February 28, 2014	NCT02074657	II	Hospital Universitario La Paz, Spain
anti-MUC1 CAR-pNK cells	Patients will receive CAR-pNK cell immunotherapy with a novel specific CAR targeting MUC1 antigen by infusion.	–	10	Hepatocellular Carcinoma;<br>Non-small Cell Lung Cancer;<br>Pancreatic Carcinoma;<br>Triple-Negative Invasive Breast Carcinoma;<br>Malignant Glioma of Brain;<br>Colorectal Carcinoma;<br>Gastric Carcinoma	July 21, 2016	NCT02839954	I/II	PersonGen BioTherapeutics (Suzhou) Co., Ltd., China
GEM/DOX + TGFBi expanded NK cells	1 x 10^8 cells/kg/dose IV on Day 12 (+ 1-2 days)	universal donor	50	Pediatric Sarcoma, Refractory;<br>Pediatric Sarcoma, Relapsed	December 2, 2022	NCT05634369	I/II	Nationwide Children's Hospital, United States
Anti-CD33 CAR-NK cells	Cells are engineered to	allogeneic NK cells (NK-92	10	Acute Myelogenous Leukemia;	October 25, 2016	NCT02944162	I/II	PersonGen BioTherape

	contain anti-CD33 attached to TCRzeta, CD28 and 4-1BB signaling domains	cell line for clinical use)		Acute Myeloid Leukemia;<br>Acute Myeloid Leukemia With Maturation;<br>Acute Myeloid Leukemia Without Maturation;<br>ANLL				utics (Suzhou) Co., Ltd., China
CAR-ITNK cells	CAR-ITNK cells will be infused over 10-15 minutes on Day 0.	–	12	B Cell Leukemia;<br>B Cell Lymphoma'<br>B-cell Acute Lymphoblastic Leukemia;<br>B-cell Lymphoma Recurrent;<br>B-cell Lymphoma Refractory	February 10, 2021	NCT04747093	I/II	Nanfang Hospital of Southern Medical University, China
UCB-NK cells	1.0-3.0 x 10^9 allogeneic UCB-NK cells	allogeneic umbilical cord blood	23	Acute Myeloid Leukemia Refractory;<br>Acute Myeloid Leukemia, Relapsed, Adult	April 15, 2020	NCT04347616	I/II	Radboud University Medical Center, Netherlands

Cetuximab + NK immunotherapy	For each procedure, 10 billion cells will be infused in 4 times	–	30	Recurrent Non-small Cell Lung Cancer	July 27, 2016	NCT02845856	I/II	Fuda Cancer Hospital, Guangzhou, China
Anti-GD2 in combination with NK cells	NK cells will be expanded over 10 days and infused in combination with anti-GD2	haploidentical donor	5	Neuroblastoma Recurrent	August 8, 2017	NCT03242603	I/II	National University Hospital, Singapore
NK cells infusion	NK cell infusion (one injection of $1x10^7$/kg CD3-CD56+ cells) after chemotherapy	haploidentical	10	Acute Myeloid Leukemia	September 20, 2013	NCT01947322	I/II	Assistance Publique - Hôpitaux de Paris, France
Irreversible electroporation + NK immunotherapy	Each treatment: 8-10 billion cells in	–	20	Recurrent Liver Carcinoma	January 2, 2017	NCT03008343	I/II	Fuda Cancer Hospital, Guangzhou, China

	all, transfuion in 3 times, i.v.							
NK cells infusion	Natural killer (NK) cell infusion Days 0 to 14 for 6 doses total.	autologous	5	Chronic Myeloid Leukemia	November 20, 2017	NCT03348033	I/II	Hospital de Clinicas de Porto Alegre, Brazil
Comparison of autogenic and allogenic NK immunotherapy	8-10 billion cells in all, transfusion in 3 times, i.v.	autogenic and allogenic	20	Malignant Solid Tumour	August 3, 2016	NCT02853903	II	Fuda Cancer Hospital, Guangzhou, China
Rituximab + NK Immunotherapy	Each time 10 billion cells, 4 times in all,i.v.	–	30	B-cell Lymphoma Recurrent	July 25, 2016	NCT02843061	I/II	Fuda Cancer Hospital, Guangzhou, China
Trastuzumab + NK immunotherapy	For each procedure, 10 billion cells will be infused for 4 times	–	30	Recurrent Breast Cancer	July 25, 2016	NCT02843126	I/II	Fuda Cancer Hospital, Guangzhou, China

Bevacizumab + NK immunotherapy	Each treatment: 8~10 billion cells in all, transfusion in 3 times, i.v.	allogeneic	45	Malignant Solid Tumour	August 5, 2016	NCT02857920	I/II	Fuda Cancer Hospital, Guangzhou, China
A Trial to Evaluate the Safety and Efficacy of oNKord®	–	Allogeneic ex vivo-generated NK cells from CD34+ umbilical cord blood progenitor cells	33	Acute Myeloid Leukemia	November 17, 2020	NCT04632316	I/II	Glycostem Therapeutics BV
High-activity Natural Killer Immunotherapy	Each treatment: 8-10 billion cells in all, transfuion in 3 times, i.v.	-	20	Melanoma	January 2, 2017	NCT03007823	I/II	Fuda Cancer Hospital, Guangzhou, China
Modified Immune Cells (AFM13-NK) +	NK cells preloaded with the bispecific	umbilical cord blood	30	Recurrent Anaplastic Large Cell Lymphoma;	August 30, 2019	NCT04074746	I/II	M.D. Anderson

Monoclonal Antibody (AFM13)	antibody AFM13 (AFM13-NK), followed by intravenous anti-CD30/CD16A monoclonal antibody AFM13			Recurrent B-Cell Non-Hodgkin Lymphoma; Recurrent Classic Hodgkin Lymphoma; Recurrent Mycosis Fungoides; Recurrent Peripheral T-Cell Lymphoma, Not Otherwise Specified; Refractory Anaplastic Large Cell Lymphoma; Refractory B-Cell Non-Hodgkin Lymphoma; Refractory Classic Hodgkin Lymphoma; Refractory Mycosis Fungoides; Refractory Peripheral T-Cell Lymphoma, Not Otherwise Specified				Cancer Center, United States
anti-CD19 CAR-NK cells	NK cells are engineered to	The allogeneic NK cells (NK-	10	Acute Lymphocytic Leukemia;	September 8, 2016	NCT02892695	I/II	PersonGen BioTherape

	contain anti-CD19 attached to TCRzeta, CD28 and 4-1BB signaling domains	92 cell line for clinical use)		Chronic Lymphocytic Leukemia; Follicular Lymphoma; Mantle Cell Lymphoma; B-cell Prolymphocytic Leukemia; Diffuse Large Lymphoma				utics (Suzhou) Co., Ltd., China
